# Understanding the role of peer group membership in reducing HIV-related risk and vulnerability among female sex workers in Karnataka, India

**DOI:** 10.1080/09540121.2012.736607

**Published:** 2013-06-09

**Authors:** Parinita Bhattacharjee, Ravi Prakash, Priya Pillai, Shajy Isac, Mohan Haranahalli, Andrea Blanchard, Maryam Shahmanesh, James Blanchard, Stephen Moses

**Affiliations:** a Karnataka Health Promotion Trust, Bangalore, India; b Department of Community Health Sciences, University of Manitoba, Winnipeg, MB, Canada; c Research Department of Infection and Population Health, University College, London, UK

**Keywords:** community mobilization, peer groups, female sex work, HIV risk and vulnerability, India

## Abstract

In Karnataka state, South India, we analyzed the role of membership in peer groups in reducing HIV-related risk and vulnerability among female sex workers (FSWs). Data from three surveys conducted in Karnataka, a behavioral tracking survey and two rounds of integrated biological and behavioral assessments (IBBAs), were analyzed. Using propensity score matching, we examined the impact of group membership on selected outcomes, including condom use, experience of violence, access to entitlements, and the prevalence of sexually transmitted infections, including HIV infection. Focus group discussions were conducted with the FSWs to better understand their perceptions regarding membership in peer groups. Peer group members participating in the IBBAs had a lower prevalence of gonorrhea and/or chlamydia (5.2 vs 9.6%, *p* <0.001), and of syphilis (8.2 vs 10.3%, *p* <0.05), compared to non-members. The average treatment effect for selected outcome measures, from the propensity score matching, showed that FSWs who were members of any peer group reported significantly less experience of violence in the past six months, were less likely to have bribed police to avoid trouble in the past six months, and were more likely to have obtained at least one formal identification document in the past five years, compared to non-members. In focus group discussions, group members indicated that they had more confidence in dealing with situations of forced sex and violence. Including community mobilization and peer group formation in the context of HIV prevention programing can reduce HIV-related risk and vulnerability among FSWs.

## Introduction

There is growing evidence that community mobilization plays a powerful role in HIV prevention programing among marginalized communities such as female sex workers (FSWs). Community mobilization, defined as a process whereby marginalized communities are mobilized or mobilize themselves to challenge power that shapes their risk, is considered as an important structural intervention in relation to risk for HIV infection (Blankenship, Biradavolu, Jena, & George, 2010). Community mobilization seeks to transform the context that increases the vulnerability of FSWs to HIV (Blankenship et al., 2010; Evans, Jana, & Lambert, 2010). Inclusion of structural interventions in HIV prevention programs is gaining acceptance, as practitioners are increasingly looking for a long-term response to the HIV epidemic (Auerbach, Parkhurst, & Caceres, 2011; Coates, Richter, & Caceres, 2008). Structural interventions aim to empower FSWs through the development of group solidarity that enables workers to collectively enforce safer norms in their interactions with clients, and to pursue collective action to improve their lives (Evans et al., 2010).

Community mobilization strategies involve a combination of activities, including consciousness raising among marginalized groups about their rights and strategies for demanding them, engaging in advocacy with stakeholders and power brokers who exercise power and control, and identifying barriers to prevention practices (Blankenship, Friedman, Dworkin, & Mantell, 2006). Community mobilization through collectivization and formation of peer groups has been used and recognized as an effective strategy to empower women. Many FSW community-based organisations (CBOs) have explicitly articulated that solidarity and unity among FSWs is an important aspect of empowerment (Cornish, Shukla, & Banerji, 2010). Membership in collectives and groups enhances their social capital, resulting in a change in the public- and self-perception of women's power (India HIV/AIDS Alliance, 2007; Halli, Ramesh, O'Neil, Moses, & Blanchard, 2006). This is reflected in their greater agency, enabling them to make decisions on issues affecting their lives. Membership in peer group collectives can improve awareness and knowledge levels of FSWs with regard to safe sex practices and promote better health-seeking behavior (Halli et al., 2006; Khandagale, Sevakari, D'Souza, Pawar, & Gilada, 2004).

The Karnataka Health Promotion Trust (KHPT) has been implementing HIV prevention programs funded by *Avahan*, the India AIDS Initiative of the Bill & Melinda Gates Foundation, in 20 districts of Karnataka, India since 2003. Details of the programmatic context have been described previously (Gurnani et al., 2011). KHPT's programs involved comprehensive outreach, education, and other services to reduce the risk of HIV and other sexually transmitted infections (STIs) by promoting behavior change and access to STI services. In addition, the program has implemented and scaled up community-based mobilization activities focusing on building individual capabilities to foster a positive perception of self, enhance self-confidence and agency among individual FSWs, and promote a collective identity to address their immediate needs, such as dealing with crises and violence. The program supports FSWs to collectively challenge structural factors underlying their risk and vulnerability, and attempts to build an enabling environment by sensitizing a range of stakeholders in and beyond the community level to address factors in the macro-level social environment that creates structural barriers to empowerment among FSWs.

Rather than treating FSWs as merely beneficiaries of prevention programing, KHPT has emphasized their active participation in all aspects of program management. Although the prevention programs were initially implemented largely by nongovernmental organizations, currently about 70% of programs have devolved program management to FSW CBOs.

Community mobilization has been shown to be an effective strategy for improving HIV preventive interventions, bringing about changes in practices, policies, and laws and reducing stigma and discrimination (Mohan, Bidappa, Moses, & Schroeder, 2010). The processes and organizational structures adopted in facilitating community mobilization may differ across contexts and districts, but follow a common set of principles and approaches that include engagement with policy makers, and addressing stigma and discrimination, violence and harassment, and social inequity (Beattie et al., 2010; Gurnani et al., 2011).

In some districts in Karnataka, FSWs have come together as members of cooperative bank structures, while in others they have organized themselves into small site-level “affinity groups,” with federation at sub-district and district levels. The community mobilization process has facilitated FSWs to come together and organize themselves under formal or informal institutional structures (collectives or peer groups), so that they can take up collective action against power imbalances and the social exclusion that disadvantage FSWs (Pillai, Bhattacharjee, Ramesh, & Isac, 2011).

In this paper, using both quantitative and qualitative methods, we contribute to the growing evidence on the importance of community mobilization in HIV prevention, by examining the relationship between FSWs’ membership in collectives or groups, and their HIV-related risk and vulnerability. We also attempt to understand the impact of group membership on violence, the enabling environment, and linkage with social entitlements.

## Methods

### Study design and sampling

This analysis involved data from three large surveys conducted in Karnataka between 2005 and 2010. These were a behavioral tracking survey (BTS), conducted in 2010 in five districts (Belgaum, Gulbarga, Gadag, Dharwad, and Solapur^1^) and two rounds of integrated biological and behavioral assessments (IBBAs), conducted in the districts of Belgaum, Bellary, Shimoga, Bangalore Urban, and Mysore, between 2005 and 2009. The five IBBA districts were chosen purposively based on Karnataka's sociocultural regions and the size of the high-risk populations, whereas the five BTS districts were purposively chosen to include intervention districts not covered by the IBBAs^2^ and representing different sociocultural regions. The first round of IBBA was carried out 7–19 months after the initiation of HIV prevention programing in each district (between January 2004 and April 2005), and the follow-up IBBA surveys were conducted 28–37 months after the baseline survey. The purpose of the BTS was to understand sexual risk behavior among FSWs, as well as issues related to community mobilization and empowerment. The IBBAs were designed to estimate the prevalence of HIV and other STIs, to understand the socioeconomic and behavioral factors associated with these infections, and to monitor trends in HIV/STI prevalence.

A probability-based sampling method was used to select the 425 FSWs in each cross-sectional survey. FSWs were sampled from various geographical sites (rural and urban) and different sex work typologies (home-based, brothel-based, lodge- or dhaba-based, and street-based) to be representative of the total population of FSWs in each district. A mapping exercise was conducted prior to the survey to identify the various typologies and geographical locations where FSWs use to solicit and carry their profession. This provided the sampling frame for the selection of the required number of FSWs. Conventional cluster sampling was used for FSWs selling sex at home, or in brothels, lodges, and dhabas, where the population of FSWs was relatively stable. A time-location cluster (TLC) sampling technique was used to draw samples of FSWs who solicited in public places (street-based). The same sampling technique was used for both IBBA and BTS surveys and hence the data from both the surveys are comparable. The detailed methodology of selection of respondents in the IBBAs has been described elsewhere (Ramesh et al., 2010; Subramanian et al., 2008; Saidel et al., 2008).

### Study population and analytical methods

This analysis is based on 1750 FSWs interviewed in the BTS and 4699 FSWs from the IBBAs (2312 in Round-1 and 2387 in Round-2). The outcomes in our analysis from the BTS were condom use with regular partner, consistent condom use with all clients/ partners, experience of physical violence (hurt, slapped, pushed, kicked, punched, choked, or burned) in past six months, forced sex in past year, enhancement to the enabling environment measured by the variable “given nothing to the police to avoid trouble in the past six months,” and access to social entitlements, using the variable “obtained an ID in the last five years.” Outcomes from IBBA data were the prevalence of gonorrhea and/or chlamydial infection, syphilis, and HIV infection. Membership in peer groups was defined as the exposure variable, and various socioeconomic and demographic characteristics were considered as independent variables.

All statistical analyses were performed using survey data analysis techniques in STATA version 10.0™ (StataCorp, College Station, TX, USA). For both data-sets, appropriate weights were used to account for the differential recruitment of FSWs by typology within districts, differential non-response rates, and differential probabilities of selection across districts. Bi-variate and multiple logistic regression analyses were used for measuring associations, and the Wald chi-square test was the statistical test used. In order to analyze the impact of membership in peer groups/collectives on outcome measures, the propensity score matching (PSM) method was used. PSM generated a set of “control” cases (individuals who were not part of any group) corresponding to “treatment” cases (individuals who belonged to a community group). Individuals who were members of any community group were matched to individuals having no membership with similar predicted probabilities (propensity score) of being a member, conditional on a set of observable characteristics.

The key assumption in this approach is that conditional on the propensity score, assignment to the treatment (member) and control (non-member) groups can be taken to be random (Rosenbaum & Rubin, 1983). If this is the case, then the difference in outcomes between treatment and control groups can be directly compared to give the effect of “treatment.” Impact was measured by the average treatment effect among those treated for selected outcome measures. This gave the difference in outcomes between the treated cases (members) and matched control cases (non-members). The statistical differences in outcome indicators between members and non-members were measured by the bootstrap standard errors for the matched sample (100 replications) in regression analysis (Rosenbaum & Rubin, 1983, 1985; Rubin, 1977).

To better understand the relationship between peer group/collective membership and project outcomes, 28 focus group discussions (FGDs) were conducted with 307 FSWs, including 157 home-based and 150 street-based FSWs in three districts (Shimoga, Bellary, and Bangalore Urban). For logistics reasons, only three districts were included in the qualitative analysis, and roughly equal numbers of peer group members and non-members participated. Each FGD was facilitated by a researcher following an FGD guideline developed for this study. In each FGD, a stratified (home- and street-based FSWs) random sample of sex workers was chosen from the registered sex workers in each district. Each FGD was conducted in *Kannada*, the local language, and recorded using a digital recorder. Following each FGD, the discussions were transcribed in English by a translator. Transcribed manuscripts from the FGDs were coded and analyzed.

All study participants were recruited on the basis of informed, voluntary consent. Institutional review boards at the University of Manitoba in Winnipeg, Canada and the St. John's Medical College and Hospital in Bangalore, India approved the study.

## Results

Of the FSWs interviewed in the BTS, 76.7% belonged to a peer group or collective (Table 1). Peer group members were significantly more likely to be older; live in the place of the interview (non-migrant); solicit at home or on the street (as opposed to brothel-based); have lower weekly client volumes (less than nine clients/week); and have had more than two years of exposure to the program. Collective membership was highest in Gadag (86.6%) and lowest in Solapur district (63.3%). Marital status, educational status, other sources of income, and duration in sex work, were not significantly associated with peer group or collective membership status.

**Table 1.** Multivariate analysis of membership in a peer group by socio-demographic and sex work characteristics: BTS, 2010.

	Membership in group/collective			
Socio-demographic and sex work factors	*N*	(%)	Adjusted OR (95% CI)	*p*-Value
*Age in completed years*				
<=24	245	66.5	Ref.	
25–29	379	73.3	1.074 (0.712, 1.618)	0.618
30–34	341	76.4	1.240 (0.805, 1.910)	0.252
35 +	774	81.6	1.505 (0.987, 2.293)	<0.05
*Educational status*				
Literate	478	75.6	Ref.	
Illiterate	1261	77.1	1.075 (0.840, 1.375)	0.273
*Marital status*				
Never married	224	75.4	Ref.	
Currently married	782	76.9	1.039 (0.734, 1.473)	0.431
Divorced/separated	733	76.8	1.036 (0.729, 1.471)	0.805
*Migration*				
Migrant	123	59.2	Ref.	
Non-migrant	1615	77.9	1.600 (1.006, 2.545)	<0.05
*Having source of income other than sex work*				
No	551	72.2	Ref.	
Yes	1186	78.7	1.118 (0.843, 1.483)	0.480
*Place of solicitation*				
Brothel/lodge/dhaba	77	53.2	Ref.	
Home/rented room	181	73.6	1.591 (0.858, 2.951)	0.112
Public place (“street”)	1016	78.6	1.999 (1.191, 3.355)	<0.01
Other	465	76.9	1.796 (1.038, 3.110)	<0.05
*Duration in sex work*				
< 2 years	189	65.4	Ref.	
2–4 years	641	74.6	0.984 (0.634, 1.526)	0.206
5–9 years	476	78.4	1.080 (0.665, 1.753)	0.172
10 + years	433	82.7	1.269 (0.740, 2.177)	<0.05
*Weekly client volume*				
10 +	263	65.6	Ref.	
5–9	452	78.6	1.931 (1.365, 2.729)	<0.01
<5	1024	78.6	1.927 (1.433, 2.591)	<0.01
*Duration of exposure to program*				
<=2 years	716	69.6	Ref.
>2 years	1006	82.5	2.042 (1.620, 2.572)	<0.01
*District*				
Belgaum	382	85.3	Ref.	
Gulbarga	374	77.4	0.563 (0.375, 0.845)	<0.01
Gadag	287	86.6	1.105 (0.678, 1.800)	0.714
Dharwad	341	71.6	0.483 (0.317, 0.736)	<0.01
Solapur	355	63.3	0.354 (0.222, 0.566)	<0.001
Totals	1739	76.7		

Notes: Analysis excluded 11 cases for which information on group membership was missing. Ref: Categories with the lowest collective membership were taken as reference category for the logistic regression model. The model was adjusted for all the socio-demographic and sex work characteristics mentioned in the table.

As shown in Table 2, sex workers participating in the BTS who were members of peer groups experienced less physical violence in the past six months compared to non-members (19.7 vs. 28.2%, *p* < 0.001). About 19.5% of sex workers who were not group members were forced to have sex in the past year, compared to 15.8% of those in a group (*p* < 0.10). About 88.5% of group members did not give bribes to police to avoid trouble in the past six months compared to 82.3% of non-members (*p* < 0.001). A higher proportion of members (67.7%) received at least one identification document (voter card/bank account/ration card) in the past five years compared to non-members (61.6%, *p* <0.05). Condom use was similar in both groups, both with regular partners and clients.

**Table 2.** Behavioral outcomes by peer group or collective membership status: BTS, 2010.

Predictor	Condom use with regular partner at last sex	Consistent condom use with all partner/clients	Experienced violence in the past six months	Beaten/forced to have sex in past one year	Did not give bribe to police to avoid trouble	Obtained any form of identification document in past five years
*Membership in peer group or collective*						
No (*N* = 409)	49.8	90.5	28.2	19.5	82.3	61.6
Yes (*N* = 1330)	54.3	91.3	19.7	15.8	88.5	67.7
Significance (χ^2^)	0.163	0.626	<0.001	0.090	<0.01	<0.05
Adjusted OR	1.250	1.071	0.700	0.837	1.460	1.227
(95% CI)^a,b^	(0.928, 1.684)	(0.707, 1.623)	(0.531, 0.922)	(0.615, 1.138)	(1.035, 2.059)	(0.955, 1.577)
*p*-Value	0.14	0.75	0.01	0.26	<0.05	0.090

Notes: *The* analysis excluded 11 cases for which information on membership was missing.

a The reference category for the logistic regression model was no membership in a peer group or collective.

b The model was adjusted for all of the socioeconomic and sex work characteristics mentioned in Table 1.

Peer group members participating in the IBBAs had a lower prevalence of gonorrhea and/or chlamydia (5.2 vs 9.6%, *p* <0.001) and of syphilis (8.2 vs 10.3%, *p* <0.05), compared to non-members (Table 3). Although the prevalence of HIV was lower among the peer group members compared to non-members, the difference was not statistically significant.

**Table 3.** Biological outcomes by peer group or collective membership status: IBBA, 2005–2008.

Predictor	Gonorrhoea and/or Chlamydia	Syphilis	HIV infection
Membership in peer group or collective			
No (*N* = 2937)	9.6	10.3	18.6
Yes (*N* = 1762)	5.2	8.2	16.9
Significance (χ^2^)	<0.001	0.05	0.17
Adjusted OR (95% CI)^a,b^	0.603 (0.468, 0.777)	0.741 (0.583, 0.940)	0.890 (0.741, 1.069)
*p*-Value	<0.001	0.01	0.11

Notes: ^a^The reference category for the logistic regression model was no membership in a peer group or collective.

b The Model was adjusted for all of the socioeconomic and sex work characteristics mentioned in Table 1.

Table 4 shows the ATT for selected outcome measures, from the propensity score matching, using both BTS and IBBA data. From the BTS data, FSWs who were members of any community group reported significantly less experience of violence in the past six months, were less likely to have bribed police to avoid trouble in the past six months, and were more likely to have obtained at least one identification document in the past five years, compared to non-members. From the IBBA data, peer group members had significantly lower prevalence of gonorrhea and/or chlamydia, and a lower prevalence of syphilis.

**Table 4.** Propensity score matching analysis: Effect of peer group or collective membership on various outcome measures, comparing FSW peer group or collective members with matched controls: BTS, 2010 and IBBA, 2005–2008.

	Membership in peer group/collective					
Outcomes	Treated (%)	Matched controls (%)	Average treatment effect ATT (adjusted effect of membership)	Bootstrap standard error	*p*-Value	Data source
Condom use with regular partner	54.7	49.7	5.0	0.0091	<0.10	BTS, 2010
Consistent condom use with all clients/partner	89.2	86.7	2.5	0.0105	–	
Experienced violence in past six months	22.2	31.7	− 9.5	0.0055	<0.001	
Beaten or forced to have sex in the past one year	17.6	23.8	− 6.2	0.0978	–	
Given nothing to police to avoid trouble in past six months	87.5	82.3	5.2	0.0051	<0.001	
Obtained any form of identification document in last five years	70.5	65.1	5.4	0.0065	<0.05	
Gonorrhoea and/or Chlamydia	5.7	10.6	− 4.9	0.0077	<0.001	IBBA, 2005–2008
Syphilis	8.4	10.3	− 1.9	0.0078	<0.05	
HIV infection	16.2	17.3	− 1.1	0.0111	–	

In focus group discussions, FSWs who were members of peer groups displayed more confidence in dealing with situations of forced sex and violence, resulting in their ability to better negotiate condom use. Their ability to counsel and convince their clients regarding condom use and to enlist the support of other sex workers in the area was cited as reasons for this confidence:

… we can phone up our friends and tell them to come for our support. We will also give him (a client) a warning that there are more community women around. (Street-based FSW, group member, Bellary)

While group members used the community network to inform their peers of a bad client and garnered their support in the event of violence, non-members lacked this support system:

Before we joined Swathi (collective), it was difficult, but now we inform the SNS (crisis management system), and the team comes and helps us solve the problem. (Home-based FSW, group member, Bangalore Urban)

Group members also negotiated situations where free sex was sought by clients better than non-members:

We first take money and sex later. (Street-based FSW, group member, Bangalore Urban)

There are clients who will not give us money even for bus charge, and some won't even give us our clothes. (Home-based FSW, non-member, Shimoga)

Whether group members or non-members, home-based FSWs were generally better equipped than street-based FSWs to handle problematic situations, as street-based FSWs practised sex work in less secure environments such as open spaces, streets, public toilets, bus stands, etc., compared to the safer environment of a home:

If there are loud voices our neighbours will come for our help. (Home-based FSW, group member, Bellary)

In our area we will resist any such attempt because we have other women for support. When we are taken out, we are helpless, and we will have to succumb, and our main aim is to get out of there alive. (Street-based FSW, member, Shimoga)

Even peer group members, especially those who were street based, expressed their helplessness in dealing with police violence. Once arrested and sent to a remand home, more often than not, they found it difficult to secure release, as only family members were permitted to release them:

I urgently needed money because my children were in a hostel. I asked a male friend for money and he took me to the Majestic area. There a policeman stopped us and accused my friend of forcibly bringing me there. He then forcibly had sex with me and stole my mobile phone. My male friend did not have sex with me. The policeman then forcibly took Rs. 350 from my male friend and left both of us with no money. (Home-based FSW, group member, Bangalore Urban)

Group members expressed less dependence on exploitative sources of credit than non-members, especially in Bangalore and Bellary districts, where the collectives had initiated micro-credit and savings schemes:

Even if we are short of money, we can always use the money saved in the group. (Home-based FSW, group member, Bellary)

In chit funds the interest is more, and if we do not pay on time, they will abuse us. (Home-based FSW, group member, Bangalore Urban)

Members also reported that alternate sources of income and credit had enabled them to refuse unprotected sex. Savings in a group emerged as a key source of funds that served two purposes: (1) savings reduced their dependency on local private financiers, thereby helping them move out of debt and (2) savings provided them with the confidence to refuse unprotected sex even for more money:

We will pay an interest penalty, but not accept free sex. (Street-based FSW, group member, Bellary)

We succumb to clients who pay more money for sex without condoms. (Street-based FSW, non-group member, Bangalore Urban)

In the focus group discussions, membership in a collective did not emerge as a factor that influenced knowledge around condoms and STIs, or to accessibility of condoms or clinical services. Both members and non-members reported not feeling guilty or immoral because of being sex workers, and asserted their right to practice sex work for a living. However, the majority of the FSWs (members and non-members) did not generally reveal their sex worker identity, even when their families were aware of it. They feared stigma and discrimination from the larger community.

## Discussion

FSWs in India face disproportionate stigma and discrimination that make them vulnerable to HIV. Altering power dynamics and reducing social exclusion can facilitate reduction in their vulnerability and risk. Community mobilization, by facilitating critical consciousness and the agency to take up collective action, can bring about structural change in power relationships (Pillai et al., 2011). Arguments and evidence for multi-level structural interventions to address HIV risk and vulnerability have grown stronger in recent years. Tools such as collective organizing and actions, which have been used by marginalized groups such as factory workers and gay men, are being increasingly used in HIV prevention programs among FSWs (Blankenship, West, Kershaw, & Biradavolu, 2008).

We have shown that there is a strong relationship between membership in groups/collectives and reduced HIV risk and vulnerability among FSWs. FSWs who are members of a collective or a group experience less violence, fewer exploitative practices (such as police bribery), and have better access to social entitlements. They also have lower rates of gonorrhea/chlamydia and syphilis. The enhanced negotiating ability of sex workers in groups may be due to either increased exposure to training and capacity building and/or improved access to credit and savings. The latter may provide financial security to decline unsafe sex with a client. Membership in groups may also create group norms for members, which encourage them to access HIV prevention services and attend health clinics regularly. FSWs who are not group members may feel more fearful, isolated, and economically dependent, and hence, may find it difficult to decline a client who offers more money for unprotected sex. Similarly, high dependence on sex work may discourage non-group members from taking time off from sex work to access HIV prevention services and attend health clinics. The focus group discussions showed that members of groups shared a sense of support and confidence, and enjoyed increased access to information about social entitlements. Further, the FGDs revealed that the benefits of collectives are experienced also by non-members, as community mobilization benefits the larger community. However, although collectivization does promote safer environments and improves the negotiation skills of FSWs, stigma and discrimination against sex workers are still common in the larger community, and that can be a restraining factor in mobilizing the community. More work needs to be done to address the issues of stigma and discrimination that sex workers face, by strengthening advocacy with policy-makers, government, and the general public.

HIV prevention programing for FSWs must go beyond condom provision and STI service promotion, to facilitate the development of a critical consciousness. This will enable FSWs to understand the structural causes of their vulnerability and marginalization, and undertake collective action against them. Prevention programs need to invest in community mobilization for empowerment of FSWs, focusing on their needs, dignity, and participation. It is important to acknowledge that there is no standard method of community mobilization. Programs should ensure that FSWs participate in developing the processes that best address their needs and suit their context.

Limitations of this study include the use of data from cross-sectional surveys, where causality cannot be ascertained. The cross-sectional designs also did not allow us to account for women leaving sex work, and the impact on STIs and HIV may be diminished if sex workers in peer groups stay in sex work longer than non-group members. In addition, group members tended to have been exposed longer to programs than non-members, which might influence their behavior, and might not have been fully adjusted for in the analysis. Finally, considerable effort and resources have been devoted over the past eight years in these districts to promote an enabling environment. These efforts could have diluted some of the effects of collectivization, as all FSWs (group members and non-members) would have benefited from the indirect effects of structural changes to the environment.

Areas for further research include exploring potential negative effects on sex workers who are not members of peer groups. It would be interesting to explore if clients are seeking sex workers who are not part of peer groups as they may have less skills of negotiation for condom use or for dealing with potentially violence situations.

In conclusion, including community mobilization and peer group formation in the context of HIV prevention programing can reduce HIV-related risk and vulnerability among FSWs. These program elements should be more widely adopted, with appropriate modifications to suit local sex work-related, political, and legal contexts.

## Notes

1. Solapur is a district in southern Maharashtra where KHPT manages HIV prevention programs, and was included in the BTS due to the high number of FSWs, and to provide a comparison with the other districts, which are all in Karnataka.

2. Belgaum was included in both the BTS and IBBAs, and was considered an important district due to the large number of FSWs and high prevalence of HIV among FSWs, as was seen in the first IBBA round in Belgaum.
